# Impact on mortality of prompt admission to critical care for deteriorating ward patients: an instrumental variable analysis using critical care bed strain

**DOI:** 10.1007/s00134-018-5148-2

**Published:** 2018-05-07

**Authors:** Steve Harris, Mervyn Singer, Colin Sanderson, Richard Grieve, David Harrison, Kathryn Rowan

**Affiliations:** 1 Critical Care Department, University College Hospital London, 235 Euston Road, London, NW1 2BU UK; 2 Wolfson Institute for Biomedical Research, University College London, The Cruciform Building, Gower Street, London, WC1E 6B UK; 3 Department of Health Services Research and Policy, London School of Hygiene and Tropical Medicine, Keppel Street, London, WC1E 7HT UK; 4 Intensive Care National Audit and Research Centre, Napier House, 24 High Holborn, London, WC1V 6AZ UK

**Keywords:** Intensive care, Deteriorating ward patient, Rapid response, Bed numbers, Occupancy, Health services research

## Abstract

**Purpose:**

To estimate the effect of prompt admission to critical care on mortality for deteriorating ward patients.

**Methods:**

We performed a prospective cohort study of consecutive ward patients assessed for critical care. Prompt admissions (within 4 h of assessment) were compared to a ‘watchful waiting’ cohort. We used critical care strain (bed occupancy) as a natural randomisation event that would predict prompt transfer to critical care. Strain was classified as low, medium or high (2+, 1 or 0 empty beds). This instrumental variable (IV) analysis was repeated for the subgroup of referrals with a recommendation for critical care once assessed. Risk-adjusted 90-day survival models were also constructed.

**Results:**

A total of 12,380 patients from 48 hospitals were available for analysis. There were 2411 (19%) prompt admissions (median delay 1 h, IQR 1–2) and 9969 (81%) controls; 1990 (20%) controls were admitted later (median delay 11 h, IQR 6–26). Prompt admissions were less frequent (*p* < 0.0001) as strain increased from low (22%), to medium (15%) to high (9%); the median delay to admission was 3, 4 and 5 h respectively. In the IV analysis, prompt admission reduced 90-day mortality by 7.4% (95% CI 1.7–18.5%, *p* = 0.117) overall, and 16.2% (95% CI 1.1–31.3%, *p* = 0.036) for those recommended for critical care. In the risk-adjust survival model, 90-day mortality was similar.

**Conclusion:**

After allowing for unobserved prognostic differences between the groups, we find that prompt admission to critical care leads to lower 90-day mortality for patients assessed and recommended to critical care.

**Electronic supplementary material:**

The online version of this article (10.1007/s00134-018-5148-2) contains supplementary material, which is available to authorized users.

## Take-home message

**Table:** 

In NHS hospitals, deteriorating ward patients referred to ICU are vulnerable: one in eight will die within a week of the referral, and half of those deaths will occur without ICU admission. While ICU admission delays are common, this study shows that prompt admission reduces mortality after allowing for both observed and unobserved differences in prognosis.	

## Introduction

Recent policy stresses the importance of identifying and responding to the deteriorating ward patient [1]. Current guidelines recommend that critical care admission should be delivered within 4 h [2]. However, supporting evidence is limited because randomised evaluation of prompt admission to critical care is deemed unethical. Yet without quantification of the benefits, it is difficult to assess the magnitude and importance of this problem.

Non-randomised evaluations are primarily confounded by treatment allocation bias [3]. Patients are prioritised on the basis of clinical severity so prompt admissions tend to have poorer prognoses. Risk adjustment will help remove this bias, but depends heavily on adequate measurement of all factors driving the decision about how to treat. In general, measured severity is an incomplete description, and often there are other end of the bed factors prompting clinicians to recommend prompt admission to critical care.

An alternative to experimental randomisation is to seek an instrument that naturally randomises patients to prompt admission or not. This natural randomisation is known as instrumental variable (IV) analysis, and has been similarly used to remove unmeasured confounding in the assessment of influenza vaccine efficacy and cardiac catheterisation [4, 5].

Here, we used critical care unit strain, measuring bed occupancy rates at the specific time of the patient’s assessment, for this purpose. This approach circumvents selection bias found in previous observational studies comparing prompt to delayed admission [6–13]. Delay can only be defined with hindsight. The bedside choice is to ‘admit now’ or to ‘watch and wait’, not to admit now or deliberately delay. A clinician neither admits the watched patient who improved nor can admit the watched patient who unexpectedly died. The delayed admissions are the subgroup of watchful-waiting controls who survive but continue to deteriorate.

A fair comparison requires prospective follow-up of all watchful-waiting controls. Our prospective study does this and also exploits the learning opportunity created by a constrained supply of critical care beds. Critical care bed provision in the UK is lower than in the majority of European (6.6 adult critical care beds versus a European average of 11.5 beds per 100,000 population) and North American health care systems [14, 15]. In settings where critical care capacity is less constrained, using strain to evaluate the effect of delay would be difficult.

We first describe the effect of critical care strain on decision-making, and the delivery of critical care for all deteriorating ward patients referred to critical care. We explore whether delays to admission engendered by high strain allow us to estimate the effect of effect of delay on patient outcome. Finally, we focus on the subgroup recommended for critical care by the bedside clinician.

## Methods

### Study design, participants and procedures

The full study protocol is available on the Intensive National Audit and Research Centre’s (ICNARC) website. In brief, the (SPOT)light study was a prospective cohort study of the deteriorating ward patient referred for assessment by critical care. The assessment had to be conducted on an inpatient ward by either a member of the critical care medical staff or the critical care outreach team (CCOT). Repeat visits and re-admissions were excluded as were patients where intensive care units (ICU) admission was either a priori refused (treatment limitation orders) or inevitable (cardiac arrests, admissions temporarily housed in theatre recovery etc.). Admissions following surgery, where delay was due to the process of care, were also excluded.

The study was registered on the National Institute of Health Research (NIHR) research portfolio (No. 9139). Hospitals were eligible for inclusion if they participated in the national clinical audit for critical care—the ICNARC case mix programme (CMP). Research teams at each hospital attended a data set familiarisation course and were given a manual of data definitions. The ICNARC clinical trials unit provided support for the study.

Reporting was via a secure online web portal that performed real-time field and record level validation. Hospitals were asked to report all consecutive ward referrals to critical care. Contemporaneous data collection was recommended, but missed referrals were sought and accepted retrospectively. We used the proportion of unplanned ward admissions to critical care in the CMP that were successfully linked to the (SPOT)light database to monitor data capture each month. Data from specific months in which data linkage rates fell below 80% were excluded from the primary analysis (but explored in sensitivity analyses). Further online validation reports were completed by all hospitals before the database was locked in September 2012. Fact and date of death were then requested from the NHS Information Service. CCOT provision was reported by participating hospitals. CMP and hospital episode statistics (HES) data were used to define critical care provision and hospital characteristics.

### Definitions

Physiology measurements at the time of the ward assessment were abstracted. From these, the ICNARC physiology score, the NHS National Early Warning Score (NEWS) and the Sequential Organ Failure Assessment (SOFA) score were calculated with missing values given zero weights as recommended [16–18]. The patient’s existing dependency at assessment was defined using the UK critical care minimum data set (CCMDS) levels of care: levels 0 and 1 are most commonly provided on normal wards while levels 2 and 3 are within high dependency (HDU) and ICU respectively [19]. The assessor was asked to recommend a future level of care, and recommendations for levels 2 or 3 were considered as recommendations for critical care admission. Prompt admission was defined as one within 4 h of ward assessment, in line with recently published UK guidelines [19].

The indicator of critical care unit strain was the difference between the maximum number of beds reported to ICNARC and the number of actively treated patients (not medically fit for discharge) occupying those beds at the time the ward patient was assessed. Units were defined as being under low, medium or high strain corresponding to having two or more, one, or zero or fewer empty beds (the last of these where strain exceeded reported capacity) respectively.

### Statistical analysis

The aim of the primary analysis was to estimate the effect of prompt critical care admission versus watchful waiting on 90-day mortality. We repeated the analysis in the subgroup recommended for critical care at the bedside assessment.

We first built orthodox proportional hazards models with risk adjustment to handle the anticipated treatment allocation bias according to those risk factors that were observed. We then built IV models in two stages. In the first stage, a selection model was constructed to predict prompt admission including the effect of strain. In the second stage, an outcome model was built replacing strain with the fitted prediction of prompt admission from the selection model. All models were adjusted for patient-level confounders including age, sex, reported referral delay, sepsis diagnosis, peri-arrest status, existing CCMDS level of care, and severity of illness using the ICNARC, SOFA and NEWS scores.

We tested for weak instruments using the Kleibergen–Paap *F* test, and used Huber–White (robust) standard errors to allow for hospital-level clustering and potential heteroscedasticity [20]. Bivariate probit IV models were used to ensure that model predictions were correctly constrained [21]. To aid interpretation, we also calculated the marginalised average treatment effect (ATE) for the population, and converted coefficients to approximate odds ratios (OR) by scaling by 1.6 [22]. Finally, we examined the sensitivity of the results to changes in the data linkage quality threshold (between 70% and 90%).

### Implementation

The study was registered with ClinicalTrials.gov (NCT01099813). The sample size was calculated to evaluate mortality increases from delay to admission using estimates from 2007 ICNARC CMP data. The target sample size was 12,075–20,125 patients referred to critical care, allowing for delays to occur in 10–40% of admissions and mortality effect sizes of 5–10%.

Categorical data were reported as counts and percentages, and continuous data as mean (SD) or median (IQR) values. Effect measures are reported with their 95% confidence intervals. Analyses were performed in R (version 3.03) except for the IV analysis which used the ivregress and biprobit commands provided in Stata (version 12.1).

## Results

Between September 2010 and December 2011, 49 hospitals (10 university-affiliated) submitted records for 435 study months. After cross-checking against national audit records, reporting was potentially incomplete for 66 (15%) months which were excluded. The primary analysis therefore included 369 study months, equivalent to a median eight study months per site (IQR 5–9) with a mean data linkage rate of 95%.

**Fig. 1:**
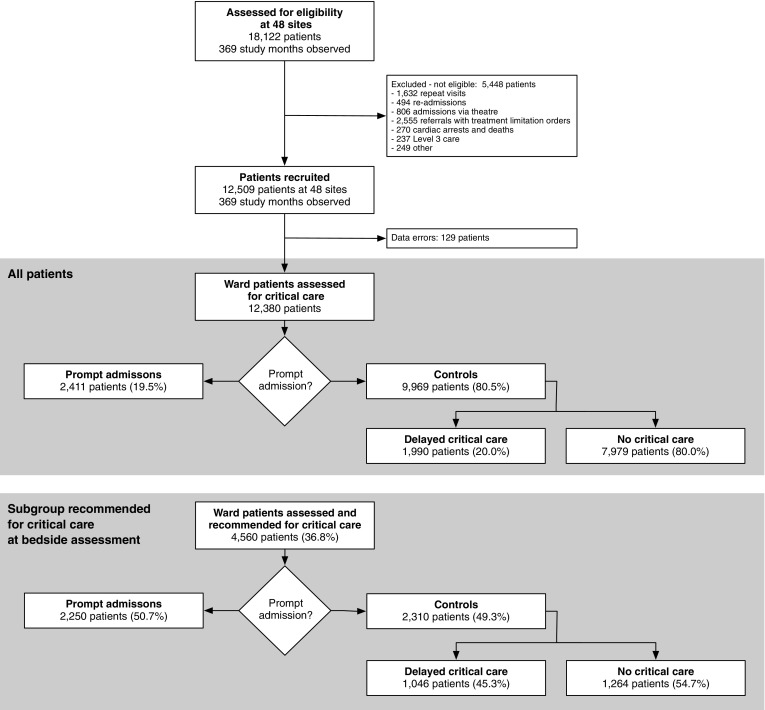
Flow diagram of patients screened: ward referrals assessed for eligibility at participating hospitals, reasons for exclusion, and admission timing following bedside assessment for all patients assessed, and for the subgroup recommended for critical care at assessment

The 369 study months captured 18,122 consecutive ward assessments. We excluded a further 2555 (14%) patients with treatment limitation orders and 1632 (9%) post-critical care follow-up visits. Timing data were unavailable for a further 129 patients. This left 12,380 patients in 48 hospitals available for analysis [a median of 222 patients (IQR 142–304) per hospital] (Fig. 1).

### Participating hospitals

There was a median of 12 critical care beds per hospital (IQR 9–18, mixed level 2 and level 3 beds) most often in a single physical location (45 hospitals). Each unit admitted a median 20 unplanned admissions (IQR 14–26) from the ward per month, representing 36% of all ICU admissions (IQR 31–43%). Critical care outreach was available 24/7 in 14 hospitals, daily but not overnight in 19 hospitals, weekday daytime only in 13 hospitals and was not offered in two hospitals.

### Patient characteristics

Table 1 shows the baseline data for all ward patients assessed. Sepsis was reported in 7586 (61%) patients; of these, the respiratory system was considered to be the source in 3851 (51%). Organ failure, defined as a SOFA score greater than one, was present in 4227 (34%) of patients. A total of 1173 patients (9%) were in respiratory failure, 2403 (19%) were in renal failure and 3629 (29%) were shocked. There was a clear correlation between physiological severity and short-term (7-day) outcome (Supplemental Fig. 1), but organ support at the time of assessment was uncommon (694 patients, 5%).

**Table 1:** Study patients and those admitted promptly

	All patients	Prompt admission	Odds ratio	(95% CI)	*p* value
	(*n* = 12,380)	(*n* = 2411)			
Age (years)					
18–39	1371 (11.1%)	258 (10.7%)			
40–59	2616 (21.1%)	567 (23.5%)	1.19	(1.01–1.41)	0.0346
60–79	5454 (44.1%)	1144 (47.4%)	1.15	(0.99–1.33)	0.0773
80–	2939 (23.7%)	442 (18.3%)	0.76	(0.64–0.90)	0.0018
Sex					
Female	5863 (47.4%)	1056 (43.8%)			
Male	6517 (52.6%)	1355 (56.2%)	1.19	(1.09–1.31)	0.0001
Reported sepsis diagnosis					
Not reported septic	4794 (38.7%)	776 (32.2%)			
Other/unspecified	1672 (13.5%)	317 (13.1%)	1.21	(1.05–1.40)	0.0093
Genitourinary	882 (7.1%)	175 (7.3%)	1.28	(1.07–1.54)	0.0077
Gastrointestinal	1181 (9.5%)	236 (9.8%)	1.29	(1.10–1.52)	0.0019
Respiratory	3851 (31.1%)	907 (37.6%)	1.60	(1.43–1.78)	< 0.0001
Referral timing					
Timely	10,814 (87.4%)	2079 (86.2%)			
Delayed	1566 (12.6%)	332 (13.8%)	1.13	(0.99–1.29)	0.0652
CCMDS level of care at visit					
Level 0	1666 (13.5%)	225 (9.3%)			
Level 1	8490 (68.6%)	1386 (57.5%)	1.25	(1.07–1.45)	0.0040
Level 2	2147 (17.3%)	779 (32.3%)	3.65	(3.09–4.30)	< 0.0001
Acute physiology scores					
ICNARC	14.0 (10.0–20.0)	18.0 (13.0–24.0)	1.09	(1.08–1.09)	< 0.0001
SOFA	3.0 (2.0–4.0)	4.0 (2.0–6.0)	1.29	(1.26–1.31)	< 0.0001
NEWS	6.0 (4.0–8.0)	8.0 (5.0–10.0)	1.19	(1.18–1.21)	< 0.0001
NEWS risk class					
None	336 (2.7%)	44 (1.8%)			
Low	3224 (26.0%)	399 (16.5%)	0.94	(0.67–1.31)	0.7039
Medium	3570 (28.8%)	529 (21.9%)	1.15	(0.83–1.61)	0.3939
High	5250 (42.4%)	1439 (59.7%)	2.51	(1.81–3.46)	< 0.0001
Reported to be peri-arrest					
No	11,815 (95.4%)	2103 (87.2%)			
Yes	565 (4.6%)	308 (12.8%)	5.53	(4.66–6.57)	< 0.0001
Visit recommendation					
Not for critical care	7820 (63.2%)	161 (6.7%)			
For critical care	4560 (36.8%)	2250 (93.3%)	46.34	(39.23–54.73)	< 0.0001
Critical care admission					
During 7-day follow-up	4401 (35.5%)	2411 (100.0%)			
Mortality					
7-day	1717 (13.9%)	500 (20.7%)	1.88	(1.68–2.11)	< 0.0001
90-day	3736 (30.2%)	885 (36.7%)	1.45	(1.32–1.59)	< 0.0001

Data are presented as mean (SD), median (IQR) or number (%). ICNARC, SOFA and NEWS refer to severity of illness scores derived from vital signs and laboratory tests. Odds ratios are calculated from univariate logistic regression for prompt admission to critical care

### Recommendation for critical care at bedside assessment

A total of 4560 (37%) patients were recommended for critical care at the bedside assessment. These patients were younger (by 1.3 years, 95% CI 0.7–1.9) and more acutely unwell (by 4.1 ICNARC physiology points, 95% CI 3.8–4.4) than those not recommended. Patients older than 80 years were less likely to be recommended for critical care (OR 0.61, 95% CI 0.53–0.71) even after risk adjustment (Supplemental Table 1).

### Prompt admission to critical care

Overall, 36% (4401 patients) were admitted to critical care in the following week rising to 72% (3296) of those recommended on assessment. The median time from assessment to admission was 3 h (IQR 1–9) overall and 2 h (IQR 1–5) for those recommended. Admissions were prompt for 19% (2411 patients) rising to 49% (2250 patients) for those with a bedside recommendation.

A total of 1636 (13%) patients died in the week following the assessment with 856 deaths (52%) following critical care admission and 780 deaths (48%) on the ward without admission. For those recommended for critical care at assessment, there were 862 deaths (19%) of which 663 (77%) received critical care before death and 199 (23%) occurred on the ward without ICU admission.

### Prompt admission and risk-adjusted mortality

For those admitted promptly, 90-day mortality was 36.7% (885 deaths) compared to 28.6% (2851) for the watchful-waiting control group. Patients who were admitted promptly had higher physiological severity scores; for example, the ICNARC physiology score was 4.4 ICNARC physiology points [95% CI 4.0–4.7] higher on average. Without risk adjustment, the proportion of patients who died prior to 90 days was higher for prompt critical care admissions with a hazard ratio (HR) of 1.42 (95% CI 1.32–1.54). With risk adjustment (Supplemental Table 3), survival was equivalent [HR 0.98 (95% CI 0.88–1.09), *p* = 0.702].

For the 4560 patients recommended for critical care, 90-day mortality was 37.2% (837 deaths) for prompt admissions versus 34.4% (794 deaths) for controls. Prompt admissions again had higher physiological severity [2.0 ICNARC physiology points (95% CI 1.6–2.5)]. Before risk adjustment, survival was worse (HR 1.12, 95% CI 1.02–1.24), but again was again equivalent after risk adjustment [HR 0.99 (95% CI 0.85–1.10), *p* = 0.852, Supplemental Table 4].

### Critical care strain

There were 10,039 (81%) bedside assessments when there were two or more empty beds on the critical care unit, 1353 (11%) when there was just one empty bed and 988 (8%) when the unit was already fully occupied (Table 2). As strain increased, the proportion of prompt admissions fell (21%, 15% and 9%, *p* < 0.0001), corresponding upward trend in the median time to admission: 3 h (IQR 1–8), 4 h (IQR 1–12) and 5 h (IQR 2–16) (*p* = 0.0009, Supplementary Fig. 2).

**Table 2:** Effects of strain on the admission pathway: recommendation for, and prompt admission to critical care, severity of illness, and outcomes stratified by critical care unit occupancy at the time of the bedside assessment

	Critical care beds			Test for trend
	≤ 0	1	≥ 2	*p* value
Patients referred	988 (8.0%)	1353 (10.9%)	10,039 (81.1%)	
Critical care				
Recommended	354 (35.8%)	471 (34.8%)	3735 (37.2%)	0.1407
Admitted	247 (25.0%)	425 (31.4%)	3775 (37.6%)	< 0.0001
Prompt admission	87 (8.8%)	196 (14.5%)	2128 (21.2%)	< 0.0001
Death without critical care	92 (9.3%)	79 (5.8%)	614 (6.1%)	0.0002
Time to critical care, hours	5.0 (2.2–15.8)	4.0 (1.0–12.0)	3.0 (1.0–8.0)	0.0009
ICNARC physiology score				
At referral	15.2 (7.1)	15.1 (7.2)	15.2 (7.2)	0.8266
Change between referral and admission	4.5 (9.2)	3.3 (9.1)	3.1 (9.2)	0.0301
Mortality				
7-day	147 (14.9%)	179 (13.2%)	1391 (13.9%)	0.6226
90-day	312 (31.6%)	417 (30.8%)	3007 (30.0%)	0.2326

Trends are tested using the Cochrane–Armitage test for categorical outcomes, and by evaluating continuous variables in a linear regression model

Strain varied by time of day, day of the week, and season; however, the relationship between prompt admission and occupancy remained even after adjustment (Supplemental Table 2), and there was a strong negative correlation between strain and prompt critical care admission (Kleibergen–Paap *F* statistic 83, *p* < 0.0001).

There was evidence that the delay in admission translated into further ongoing physiological deterioration (ICNARC physiology scores increased by 3.1, 3.3 and 4.5 points respectively, *p* = 0.03). Regardless of timing, the overall probability of receiving critical care also fell (38%, 32% and 25% for assessments during times of low, medium and high strain, *p* < 0.0001).

Within the subgroup recommended, the proportion of prompt admissions also fell as strain increased (53%, 38% and 23%, *p* < 0.0001) and median time to admission increased: 2 h (IQR 1–4), 3 h (IQR 1–6) and 4 h (IQR 2–9) (*p* < 0.0001). The proportion of patients managed without critical care increased from 25%, to 33%, to 50% during low, medium and high strain periods (Supplemental Table 5).

### Critical care strain and mortality

**Fig. 2:**
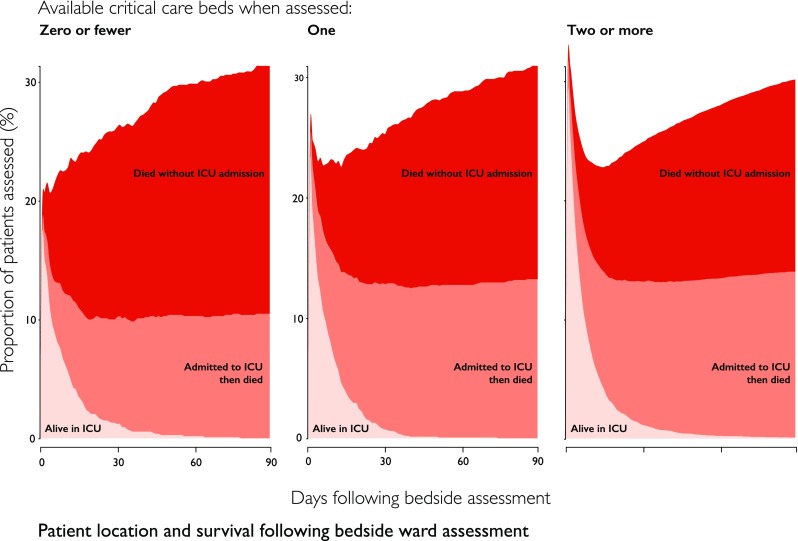
Patient disposition over time following bedside assessment: proportion of patients who are alive in critical care, who died within or following a critical care admission, or who died without admission to critical care by critical care unit strain at the time of the bedside assessment

The 90-day mortality was 30.0% (3007 deaths), 30.8% (417 deaths) and 31.6% (312 deaths) for deteriorating ward patients assessed at times of low, medium and high critical care strain respectively (Fig. 2). Using the change in mortality driven by critical care strain, the instrumental variable model estimated a reduction in 90-day mortality of 7.4% (95% CI 1.7–18.5%, *p* = 0.117) which was equivalent to an odds ratio of 0.68 (95% CI 0.42–1.10, Table 3 and Supplemental Table 6).

**Table 3:** Instrumental variable model for the effect of prompt admission on 90-day mortality: for all patients, and for the subgroup with recommended to critical care at the bedside assessment

	All patients		Recommended for critical care	
	Odds ratio	*p* value	Odds ratio	*p* value
Visiting timing				
Winter	1.02 (0.93–1.11)	0.734	0.98 (0.85–1.14)	0.816
Weekend (Saturday–Sunday)	1.02 (0.94–1.12)	0.624	1.13 (0.98–1.30)	0.083
Out-of-hours (7 p.m.–7 a.m.)	0.95 (0.87–1.04)	0.285	1.05 (0.90–1.23)	0.553
Age (per year)				
< 80 years	1.03 (1.03–1.03)	< 0.001	1.02 (1.02–1.03)	< 0.001
≥ 80 years	1.02 (1.01–1.04)	0.004	1.03 (1.00–1.06)	0.087
Male sex	1.09 (1.00–1.17)	0.043	1.09 (0.96–1.23)	0.183
Reported sepsis diagnosis				
Not septic				
Unspecified sepsis	1.06 (0.93–1.20)	0.375	1.02 (0.83–1.26)	0.834
Genitourinary sepsis	0.58 (0.48–0.69)	< 0.001	0.51 (0.38–0.68)	< 0.001
Abdominal sepsis	0.84 (0.73–0.97)	0.020	0.85 (0.67–1.06)	0.147
Chest sepsis	1.24 (1.13–1.37)	< 0.001	1.31 (1.12–1.53)	0.001
Level of care at time of visit				
Level 0	1.04 (0.90–1.20)	0.629	1.09 (0.84–1.41)	0.525
Level 1				
Level 2	0.95 (0.84–1.08)	0.469	0.90 (0.76–1.06)	0.220
Delayed referral to critical care	1.00 (0.89–1.13)	0.950	0.94 (0.79–1.13)	0.517
Reported to be peri-arrest	0.95 (0.78–1.15)	0.589	1.08 (0.86–1.34)	0.517
Acute physiology score				
NEWS	1.06 (1.05–1.08)	< 0.001	1.06 (1.04–1.09)	< 0.001
ICNARC	1.03 (1.02–1.03)	< 0.001	1.02 (1.01–1.03)	< 0.001
SOFA	1.14 (1.12–1.17)	< 0.001	1.13 (1.09–1.17)	< 0.001
Level of care recommended				
Level 0	1.01 (0.83–1.23)	0.923		
Level 1			Reference	
Level 2	1.29 (1.02–1.62)	0.034		
Level 3	1.76 (1.18–2.62)	0.006	1.53 (1.17–2.02)	0.002
Prompt admission (within 4 h)	0.68 (0.42–1.10)	0.118	0.46 (0.22–0.96)	0.036

The coefficients from the underlying bivariate probit model have been scaled by 1.6 to give OR. Age was entered into the model using a linear spline with a knot at 80 years to account for the age bias in the selection model

Among those recommended for critical care, unadjusted 90-day mortality was 35.0% (1307 deaths), 41.8% (197 deaths) and 35.9% (127 deaths) for ward assessments at times of low, medium and high strain. For this subgroup, the average reduction in 90-day mortality for prompt admission was 16.2% (95% CI 1.1–31.3%, *p* = 0.036) equivalent to an odds ratio of 0.46 (95% CI 0.22–0.96, *p* = 0.036).

## Discussion

This prospective cohort study describes the outcomes of more than 12,000 ward patients assessed by critical care teams in 48 acute NHS hospitals. Delay to admission is common. Those patients admitted promptly to critical care are manifestly more unwell, with a higher unadjusted mortality (37% vs 29%). Risk adjustment for observed severity is unable to show a benefit for a prompt admission strategy. However, we argue that unobserved differences in baseline risk are part of a clinician’s bedside assessment, and our risk adjustment is likely to be incomplete.

We exploit the random variation in critical care strain to additionally allow for unobserved differences in baseline severity. We show that prompt critical care admission is less likely for patients referred during times of strain. Using this natural experimental set-up, we found that prompt admission reduces 90-day mortality without reaching statistical significance. For the subgroup recommended for critical care, the estimated reduction in 90-day mortality is larger and statistically significant. There is a large amount of uncertainty in the precise estimate of these effects, but the weight of evidence favours a prompt admission strategy, especially where the bedside assessor recommends critical care.

The prompt admission strategy might confer a survival advantage in two distinct ways: explicitly be delivering critical care to a particular patient more promptly; or implicitly, by increasing the opportunity for critical care in the population. Of note, half of the deaths in the first week occur without admission to critical care, and death without critical care is more common when strain is greater.

It is not easy to separate out these two effects. Although previous studies compare the prompt to the ‘delayed’ admission, the delay is defined with hindsight which creates exclusion and survivor bias [6–13]. The real bedside decision is to ‘admit now’ or to ‘watch and wait’. Direct patient-level randomisation to evaluate a ‘prompt’ versus a ‘watchful waiting’ strategy is ethically challenging. Recent attempts have resorted to cluster randomisation of a strategy to increase referrals to critical care (specifically in the elderly and focussing on access not timing) [23]. This increased access but also increased severity of illness leading to the same issues with adjusting for unobserved differences in baseline severity [24].

Our attempt to minimise treatment selection bias (confounding by indication) relies on the assumption that critical strain acts as a natural randomisation event. We defend this assumption in three ways. Firstly, critical care occupancy at a specific time is unpredictable. It is the net result of all the decisions that lead to admission, discharge and death in a complex, maybe chaotic, system. Secondly, we have included controls where occupancy might be associated with baseline severity (i.e. increased strain during the winter). Thirdly, although strain might translate as a crowding effect thereby impairing the quality of care within the ICU, the evidence for this is conflicting [25, 26]. Moreover, crowding could not possibly affect the outcomes of the two thirds of patients never admitted to critical care. We additionally observed that patients assessed when there were no available beds deteriorated more prior to admission. This suggests a causal pathway that is independent of any effect of crowding after admission.

An important limitation of IV analyses is that they are notoriously weak, and, without very large sample sizes, there is a risk of not detecting a true difference when one exists [27, 28]. This may be understood if we consider IV analysis as a randomised controlled trial but with poor compliance. Critical care strain (at the time of the bedside assessment) is the random coin toss and prompt admission is the treatment randomised. However, clinical teams may find ways to deliver prompt admission even at times of high strain (perhaps by accelerating discharge or flexing staffing). We saw 92 patients admitted promptly even though there were no beds at the instant of bedside assessment. Imperfect compliance requires either large samples or large effect sizes to achieve significance. Of note, our effect size was larger for the most unwell patients, and only then achieved significance at the 5% threshold.

Our finding of harm from reduced or delayed access to critical care does not stand in isolation. At a patient level, reducing exposure to critical care by premature discharge rather than delayed admission has also increased mortality [29]. At a population level, expanding critical care capacity through an increase in funding during health service reforms is similarly associated with an improvement in outcomes [30]. The one previous study (five hospitals, 749 patients) of prompt admission to critical care that also used the watchful-waiting cohort as controls also found benefit for prompt admission [31].

We also need to acknowledge that in an observational study of this size, there are limitations in the quality of the data recorded. We did not perfectly capture all admissions to critical care in the study database, and we must assume that a proportion of referrals were also missed. However, we tested our findings by raising threshold for judging data capture to 90% so that the median proportion of eligible admissions was 97%. We found no consistent difference in any result other than a fall in precision as the quality threshold increased, and the sample size inevitably fell.

Aspects of the study stand independent of these limitations. Regardless of the effect of prompt admission to critical care, we have identified a cohort of hospital patients at very high risk. This risk is heavily front-loaded, and the window for intervention is short. The bedside assessment is an effective but imperfect triage tool, as the mortality in those initially refused admission is high. Given that we already excluded patients with treatment limitations, it is of concern that nearly half of these early deaths occur without a trial of critical care.

A substantial proportion of patients recommended for critical care are not offered a bed, and this proportion increases when capacity is limited. Although expanding critical care bed numbers may help, supply quickly saturates this expensive resource [32]. Given that the benefit of prompt critical care is unlikely to be equal for all referrals, the challenge now is to better prioritise. Interestingly, this same approach has also been highlighted as crucial to both patients and the public [33].

## Electronic supplementary material

Below is the link to the electronic supplementary material.

Supplementary material 1 (DOCX 55 kb)

Supplementary material 2 (DOCX 7702 kb)
