# Correction to: Impact on mortality of prompt admission to critical care for deteriorating ward patients: an instrumental variable analysis using critical care bed strain

**DOI:** 10.1007/s00134-018-5254-1

**Published:** 2018-06-08

**Authors:** Steve Harris, Mervyn Singer, Colin Sanderson, Richard Grieve, David Harrison, Kathryn Rowan

**Affiliations:** 1 Critical Care Department, University College Hospital London, 235 Euston Road, London, NW1 2BU UK; 2 Wolfson Institute for Biomedical Research, University College London, The Cruciform Building, Gower Street, London, WC1E 6B UK; 3 Department of Health Services Research and Policy, London School of Hygiene and Tropical Medicine, Keppel Street, London, WC1E 7HT UK; 4 Intensive Care National Audit and Research Centre, Napier House, 24 High Holborn, London, WC1V 6AZ UK

## Correction to: Intensive Care Med 10.1007/s00134-018-5148-2

This article was originally published under a CC BY-NC 4.0 license, but has now been made available under a CC BY 4.0 license. The PDF and HTML versions of the paper have been modified accordingly.

**Open access**

This article is distributed under the terms of the Creative Commons Attribution 4.0 International License (http://creativecommons.org/licenses/by/4.0/), which permits unrestricted use, distribution, and reproduction in any medium, provided you give appropriate credit to the original author(s) and the source, provide a link to the Creative Commons license, and indicate if changes were made.

## Electronic supplementary material

Below is the link to the electronic supplementary material.

Supplementary material 1 (DOCX 28 kb)

