# Supplementary material for: Impact on mortality of prompt admission to critical care for deteriorating ward patients: an instrumental variable analysis using critical care bed strain
Source: Intensive Care Med. 2018 May 7;44(5):606–15. doi: 10.1007/s00134-018-5148-2 (PMC6006241; doi:10.1007/s00134-018-5148-2)
Supplement: Supplementary file 1 — Supplementary material 1 (DOCX 55 kb) [file 134_2018_5148_MOESM1_ESM.docx]

# Supplemental Tables

## Supplemental Table 1

|  | Odds | Ratio | p-value |  |
| --- | --- | --- | --- | --- |
|  |  |  |  |  |
| **Hospital characteristics** |  |  |  |  |
| Teaching hospital | 0.94 | (0.55--1.62) | 0.835 |  |
| Overnight admissions (1000's) | 0.99 | (0.98--1.00) | 0.023 | * |
| Emergency casemix (%) | 1.02 | (0.98--1.05) | 0.333 |  |
| Critical care beds | 1.00 | (0.98--1.03) | 0.770 |  |
| CMP admissions per bed per month | 1.03 | (0.83--1.28) | 0.790 |  |
| Ward referrals to ICU (per 1,000 hosp. adm.) | 0.94 | (0.91--0.97) | <0.001 | *** |
| CCOT shift pattern |  |  |  |  |
| None | 2.44 | (0.90--6.63) | 0.081 |  |
| Less than 7 days | 1.35 | (0.80--2.28) | 0.260 |  |
| 7 days/week | 1.66 | (1.02--2.70) | 0.043 | * |
| 24 hrs/day 7 days/week | Reference |  |  |  |
|  |  |  |  |  |
| **Visit timing** |  |  |  |  |
| Out-of-hours (7pm – 7am) | 1.33 | (1.20--1.48) | <0.001 | *** |
| Weekend (Saturday – Sunday) | 1.12 | (1.01--1.25) | 0.034 | * |
| Winter (Dec – Mar) | 1.23 | (1.09--1.38) | 0.001 | *** |
|  |  |  |  |  |
| Available critical care beds |  |  |  |  |
| ≥2 | Reference |  |  |  |
| 1 | 0.97 | (0.82--1.15) | 0.717 |  |
| ≤0 | 0.96 | (0.78--1.17) | 0.675 |  |
|  |  |  |  |  |
| **Patient characteristics** |  |  |  |  |
| Age (years) |  |  |  |  |
| 18—39 | 1.03 | (0.86--1.22) | 0.750 |  |
| 40—59 | Reference |  |  |  |
| 60—79 | 0.92 | (0.82--1.05) | 0.213 |  |
| 80— | 0.61 | (0.53--0.71) | <0.001 | *** |
|  |  |  |  |  |
| Male sex | 0.99 | (0.90--1.09) | 0.792 |  |
| Reported sepsis diagnosis |  |  |  |  |
| Not septic | Reference |  |  |  |
| Unspecified sepsis | 1.14 | (0.98--1.33) | 0.080 |  |
| Genitu-urinary sepsis | 1.20 | (0.99--1.46) | 0.062 |  |
| Abdominal sepsis | 1.25 | (1.05--1.47) | 0.010 | ** |
| Chest sepsis | 1.36 | (1.21--1.52) | <0.001 | *** |
|  |  |  |  |  |
| Delayed referral to critical care | 1.08 | (0.93--1.25) | 0.310 |  |
|  |  |  |  |  |
| Level of care at time of visit |  |  |  |  |
| Level 0 | 0.53 | (0.45--0.63) | <0.001 | *** |
| Level 1 | Reference |  |  |  |
| Level 2 | 20.49 | (17.52--23.97) | <0.001 | *** |
|  |  |  |  |  |
| Reported to be peri-arrest | 4.83 | (3.75--6.22) | <0.001 | *** |
| ICNARC physiology score | 1.07 | (1.06--1.08) | <0.001 | *** |

**Recommendation for critical care at bedside assessment:** Adjusted odds ratios for hospital, timing and patient characteristics associated with a recommendation for critical care being made at the bedside assessment

## Supplemental Table 2

|  | Odds | Ratio | p-value |  |
| --- | --- | --- | --- | --- |
|  |  |  |  |  |
| **Hospital characteristics** |  |  |  |  |
| Teaching hospital | 0.89 | (0.51--1.56) | 0.688 |  |
| Overnight admissions (1000's) | 0.99 | (0.98--1.00) | 0.138 |  |
| Emergency casemix (%) | 1.03 | (0.99--1.06) | 0.113 |  |
| Critical care beds | 1.02 | (0.99--1.05) | 0.292 |  |
| CMP admissions per bed per month | 1.31 | (1.04--1.64) | 0.019 | * |
| Ward referrals to ICU (per 1,000 hosp. adm.) | 0.95 | (0.92--0.98) | 0.004 | ** |
| CCOT shift pattern |  |  |  |  |
| None | 4.10 | (1.45--11.59) | 0.008 | ** |
| Less than 7 days | 1.25 | (0.73--2.16) | 0.420 |  |
| 7 days/week | 1.44 | (0.86--2.40) | 0.162 |  |
| 24 hrs/day 7 days/week | Reference |  |  |  |
|  |  |  |  |  |
| **Visit timing** |  |  |  |  |
| Out-of-hours (7pm – 7am) | 1.82 | (1.64--2.03) | <0.001 | *** |
| Weekend (Saturday – Sunday) | 1.16 | (1.03--1.30) | 0.012 | * |
| Winter (Dec – Mar) | 0.97 | (0.85--1.10) | 0.592 |  |
|  |  |  |  |  |
| Available critical care beds |  |  |  |  |
| ≥2 | Reference |  |  |  |
| 1 | 0.64 | (0.53--0.78) | <0.001 | *** |
| ≤0 | 0.37 | (0.28--0.48) | <0.001 | *** |
|  |  |  |  |  |
| **Patient characteristics** |  |  |  |  |
| Age (years) |  |  |  |  |
| 18—39 | 1.00 | (0.83--1.20) | 0.985 |  |
| 40—59 | Reference |  |  |  |
| 60—79 | 0.83 | (0.73--0.94) | 0.005 | ** |
| 80— | 0.56 | (0.48--0.65) | <0.001 | *** |
|  |  |  |  |  |
| Male sex | 1.10 | (0.99--1.22) | 0.063 |  |
| Reported sepsis diagnosis |  |  |  |  |
| Not septic | Reference |  |  |  |
| Unspecified sepsis | 1.08 | (0.92--1.27) | 0.361 |  |
| Genitu-urinary sepsis | 1.15 | (0.93--1.42) | 0.194 |  |
| Abdominal sepsis | 1.04 | (0.87--1.26) | 0.643 |  |
| Chest sepsis | 1.24 | (1.09--1.40) | 0.001 | *** |
|  |  |  |  |  |
| Delayed referral to critical care | 0.98 | (0.84--1.15) | 0.845 |  |
|  |  |  |  |  |
| Level of care at time of visit |  |  |  |  |
| Level 0 | 0.62 | (0.51--0.76) | <0.001 | *** |
| Level 1 | Reference |  |  |  |
| Level 2 | 2.80 | (2.45--3.20) | <0.001 | *** |
|  |  |  |  |  |
| Reported to be peri-arrest | 4.12 | (3.37--5.05) | <0.001 | *** |
| ICNARC physiology score | 1.06 | (1.05--1.07) | <0.001 | *** |

**Prompt admission to critical care at bedside assessment:** Adjusted odds ratios for hospital, timing and patient characteristics associated with a prompt admission (within 4 hours) to critical care following the bedside assessment

## Supplemental Table 3

|  | Hazard ratio | (95%CI) | p-value |
| --- | --- | --- | --- |
| Age (per year) | 1.03 | (1.02--1.03) | <0.001 |
| Male sex | 1.04 | (0.98--1.12) | 0.211 |
| Reported sepsis diagnosis |  |  |  |
| Not septic | Reference |  |  |
| Unspecified sepsis | 1.02 | (0.92--1.14) | 0.696 |
| Genitu-urinary sepsis | 0.59 | (0.50--0.69) | <0.001 |
| Abdominal sepsis | 0.89 | (0.79--1.02) | 0.089 |
| Chest sepsis | 1.17 | (1.08--1.27) | <0.001 |
| Level of care at time of visit |  |  |  |
| Level 0 | 1.03 | (0.92--1.17) | 0.586 |
| Level 1 | Reference |  |  |
| Level 2 | 1.00 | (0.91--1.10) | 0.990 |
| Delayed referral to critical care | 1.00 | (0.91--1.10) | 0.993 |
| Reported to be peri-arrest | 0.94 | (0.81--1.10) | 0.453 |
| Acute physiology score |  |  |  |
| NEWS | 1.06 | (1.05--1.07) | <0.001 |
| ICNARC | 1.02 | (1.02--1.03) | <0.001 |
| SOFA | 1.12 | (1.10--1.14) | <0.001 |
| Level of care recommended |  |  |  |
| Level 0 | 0.98 | (1.05--1.07) | <0.001 |
| Level 1 | Reference |  |  |
| Level 2 | 1.11 | (1.01--1.22) | 0.036 |
| Level 3 | 1.23 | (1.06--1.43) | 0.006 |
| Admitted to critical care within 4 hours | 0.98 | (0.88--1.09) | 0.702 |

**Association between patient factors and 90-day survival**: Hazard ratios for 90-day survival

## Supplemental Table 4

|  | Hazard ratio | (95%CI) | p-value |
| --- | --- | --- | --- |
| Age (per year) | 1.02 | (1.02--1.03) | <0.001 |
| Male sex | 1.03 | (0.93--1.14) | 0.520 |
| Reported sepsis diagnosis |  |  |  |
| Not septic | Reference |  |  |
| Unspecified sepsis | 1.02 | (0.87--1.21) | 0.783 |
| Genitu-urinary sepsis | 0.52 | (0.40--0.66) | <0.001 |
| Abdominal sepsis | 0.93 | (0.77--1.12) | 0.448 |
| Chest sepsis | 1.20 | (1.06--1.36) | 0.004 |
| Level of care at time of visit |  |  |  |
| Level 0 | 1.02 | (0.84--1.24) | 0.846 |
| Level 1 | Reference |  |  |
| Level 2 | 1.00 | (0.90--1.11) | 0.990 |
| Delayed referral to critical care | 0.97 | (0.85--1.12) | 0.688 |
| Reported to be peri-arrest | 1.01 | (0.86--1.20) | 0.870 |
| Acute physiology score |  |  |  |
| NEWS | 1.06 | (1.04--1.08) | <0.001 |
| ICNARC | 1.02 | (1.01--1.03) | <0.001 |
| SOFA | 1.10 | (1.07--1.13) | <0.001 |
| Level of care recommended |  |  |  |
| Level 2 | Reference |  |  |
| Level 3 | 1.00 | (1.00--1.00) |  |
| Admitted to critical care within 4 hours | 0.99 | (0.89--1.10) | 0.852 |

**Association between patient factors and 90-day survival for the subgroup of patients recommended for critical care at the bedside assessment**: Hazard ratios for 90-day survival

## Supplemental Table 5

|  |  |  | Critical care beds | |  |  | Test for trend |
| --- | --- | --- | --- | --- | --- | --- | --- |
|  | ≤ 0 | | 1 | | ≥2 | | (p value) |
| **Patients referred** | 354 | (7.8%) | 471 | (10.3%) | 3735 | (81.9%) |  |
| **Critical care** |  |  |  |  |  |  |  |
| Admitted | 178 | (50.3%) | 318 | (67.5%) | 2810 | (75.2%) | <0.0001 |
| Prompt admission | 83 | (23.4%) | 180 | (38.2%) | 1987 | (53.2%) | <0.0001 |
| Death without critical care | 32 | (9.0%) | 19 | (4.0%) | 153 | (4.1%) | 0.0001 |
| **Time to critical care (hours)** | 4.0 | (2.0--9.0) | 3.0 | (1.0--6.0) | 2.0 | (1.0--4.0) | <0.0001 |
| **ICNARC physiology score** |  |  |  |  |  |  |  |
| at referral | 17.9 | (7.2) | 18.2 | (7.6) | 17.8 | (7.7) | 0.4061 |
| change between referral and admission | 4.2 | (9.1) | 3.0 | (9.4) | 2.6 | (9.1) | 0.0164 |
| **Mortality** |  |  |  |  |  |  |  |
| 7 day | 71 | (20.1%) | 101 | (21.4%) | 720 | (19.3%) | 0.4330 |
| 90 day | 127 | (35.9%) | 197 | (41.8%) | 1307 | (35.0%) | 0.1243 |

**Effects of strain on the admission pathway for the subgroup recommended for critical care:** prompt admission to critical care, severity of illness, and outcomes stratified by critical care unit occupancy at the time of the bedside assessment. Trends are tested using the Cochrane-Armitage test for categorical outcomes, and by evaluating continuous variables in a linear regression model.

## Supplemental Table 6

|  | All patients |  |  |  | Recommended for critical care |  |
| --- | --- | --- | --- | --- | --- | --- |
|  | Probit coefficient | (95%CI) | p-value | Probit coefficient | (95%CI) | p-value |
| Age (per year) |  |  |  |  |  |  |
| < 80 years | 0.02 | (0.02 to 0.02) | <0.001 | 0.02 | (0.01 to 0.02) | <0.001 |
| ≥ 80 years | 0.01 | (0.00 to 0.02) | 0.004 | 0.02 | (-0.00 to 0.04) | 0.079 |
| Male sex | 0.05 | (0.00 to 0.10) | 0.041 | 0.05 | (-0.02 to 0.13) | 0.176 |
| Reported sepsis diagnosis |  |  |  |  |  |  |
| Not septic | Reference |  |  | Reference |  |  |
| Unspecified sepsis | 0.04 | (-0.04 to 0.12) | 0.373 | 0.01 | (-0.12 to 0.14) | 0.840 |
| Genitu-urinary sepsis | -0.35 | (-0.46 to -0.24) | <0.001 | -0.43 | (-0.61 to -0.25) | <0.001 |
| Abdominal sepsis | -0.11 | (-0.20 to -0.02) | 0.020 | -0.11 | (-0.25 to 0.04) | 0.143 |
| Chest sepsis | 0.14 | (0.08 to 0.20) | <0.001 | 0.17 | (0.07 to 0.27) | 0.001 |
| Level of care at time of visit |  |  |  |  |  |  |
| Level 0 | 0.02 | (-0.07 to 0.11) | 0.636 | 0.05 | (-0.11 to 0.21) | 0.541 |
| Level 1 | Reference |  |  | Reference |  |  |
| Level 2 | -0.03 | (-0.11 to 0.05) | 0.460 | -0.06 | (-0.16 to 0.04) | 0.243 |
| Delayed referral to critical care | 0.00 | (-0.07 to 0.08) | 0.944 | -0.04 | (-0.15 to 0.07) | 0.517 |
| Reported to be peri-arrest | -0.03 | (-0.15 to 0.09) | 0.589 | 0.04 | (-0.10 to 0.18) | 0.548 |
| Acute physiology score |  |  |  |  |  |  |
| NEWS | 0.04 | (0.03 to 0.05) | <0.001 | 0.04 | (0.02 to 0.05) | <0.001 |
| ICNARC | 0.02 | (0.01 to 0.02) | <0.001 | 0.01 | (0.01 to 0.02) | <0.001 |
| SOFA | 0.08 | (0.07 to 0.10) | <0.001 | 0.08 | (0.06 to 0.10) | <0.001 |
| Level of care recommended |  |  |  |  |  |  |
| Level 0 | 0.00 | (-0.12 to 0.13) | 0.950 |  |  |  |
| Level 1 | Reference |  |  |  |  |  |
| Level 2 | 0.16 | (0.01 to 0.31) | 0.033 | Reference | Reference |  |
| Level 3 | 0.36 | (0.10 to 0.62) | 0.006 | 0.26 | (0.09 to 0.43) | 0.003 |
| Admitted to critical care within 4 hours | -0.25 | (-0.56 to 0.05) | 0.107 | -0.45 | (-0.90 to -0.00) | 0.049 |

**Bivariate probit model for the effect of prompt admission on 90-day mortality** for all patients, and for the subgroup with recommended to critical care at the bedside assessment.

## Supplemental Table 7

|  | Patients recommended | | Prompt admission | | Odds ratio | (95%CI) | p value |
| --- | --- | --- | --- | --- | --- | --- | --- |
|  | (n=4560) |  | (n=2250) |  |  |  |  |
| Age (years) |  |  |  |  |  |  |  |
| 18—39 | 484 | (10.6%) | 243 | (10.8%) |  |  |  |
| 40—59 | 1029 | (22.6%) | 527 | (23.4%) | 1.04 | (0.84--1.29) | 0.7145 |
| 60—79 | 2157 | (47.3%) | 1068 | (47.5%) | 0.97 | (0.80--1.18) | 0.7828 |
| 80— | 890 | (19.5%) | 412 | (18.3%) | 0.85 | (0.68--1.07) | 0.1654 |
| Sex |  |  |  |  |  |  |  |
| Female | 2068 | (45.4%) | 990 | (44.0%) |  |  |  |
| Male | 2492 | (54.6%) | 1260 | (56.0%) | 1.11 | (0.99--1.25) | 0.0706 |
| Reported sepsis diagnosis |  |  |  |  |  |  |  |
| Not reported septic | 1453 | (31.9%) | 715 | (31.8%) |  |  |  |
| Other/unspecified | 616 | (13.5%) | 299 | (13.3%) | 0.97 | (0.81--1.18) | 0.7806 |
| Genito-urinary | 319 | (7.0%) | 168 | (7.5%) | 1.15 | (0.90--1.46) | 0.2638 |
| Gastrointestinal | 476 | (10.4%) | 217 | (9.6%) | 0.86 | (0.70--1.06) | 0.1703 |
| Respiratory | 1696 | (37.2%) | 851 | (37.8%) | 1.04 | (0.90--1.20) | 0.5880 |
| Referral timing |  |  |  |  |  |  |  |
| Timely | 3894 | (85.4%) | 1938 | (86.1%) |  |  |  |
| Delayed | 666 | (14.6%) | 312 | (13.9%) | 0.89 | (0.75--1.05) | 0.1635 |
| CCMDS Level of Care at visit |  |  |  |  |  |  |  |
| Level 0 | 320 | (7.0%) | 205 | (9.1%) |  |  |  |
| Level 1 | 2336 | (51.2%) | 1268 | (56.4%) | 0.67 | (0.52--0.85) | 0.0010 |
| Level 2 | 1887 | (41.4%) | 763 | (33.9%) | 0.38 | (0.30--0.49) | <0.0001 |
| Acute physiology scores |  |  |  |  |  |  |  |
| ICNARC | 17.0 | (12.0--23.0) | 19.0 | (13.0--24.0) | 1.04 | (1.03--1.04) | <0.0001 |
| SOFA | 4.0 | (2.0--5.0) | 4.0 | (2.0--6.0) | 1.11 | (1.08--1.14) | <0.0001 |
| NEWS | 7.0 | (5.0--9.0) | 8.0 | (5.0--10.0) | 1.06 | (1.04--1.08) | <0.0001 |
| NEWS Risk Class |  |  |  |  |  |  |  |
| None | 70 | (1.5%) | 41 | (1.8%) |  |  |  |
| Low | 788 | (17.3%) | 363 | (16.1%) | 0.60 | (0.37--0.99) | 0.0463 |
| Medium | 1109 | (24.3%) | 481 | (21.4%) | 0.54 | (0.33--0.88) | 0.0142 |
| High | 2593 | (56.9%) | 1365 | (60.7%) | 0.79 | (0.49--1.27) | 0.3278 |
| Reported to be peri-arrest |  |  |  |  |  |  |  |
| No | 4105 | (90.0%) | 1947 | (86.5%) |  |  |  |
| Yes | 455 | (10.0%) | 303 | (13.5%) | 2.21 | (1.80--2.71) | <0.0001 |
| Critical care admission |  |  |  |  |  |  |  |
| During 7-day follow-up | 3296 | (72.3%) | 2250 | (100.0%) |  |  |  |
| Mortality |  |  |  |  |  |  |  |
| 7-day | 892 | (19.6%) | 475 | (21.1%) | 1.21 | (1.05--1.41) | 0.0093 |
| 90-day | 1631 | (35.8%) | 837 | (37.2%) | 1.13 | (1.00--1.28) | 0.0464 |

**Patients recommended for critical care and those then admitted promptly**: Data are presented as mean (SD), median (IQR) or number (%). ICNARC, SOFA, and NEWS refer to severity of illness scores derived from vital signs, and laboratory tests. Odds ratios are calculated from univariate logistic regression for prompt admission to critical care.
