# Supplementary material for: Impact on mortality of prompt admission to critical care for deteriorating ward patients: an instrumental variable analysis using critical care bed strain
Source: Intensive Care Med. 2018 May 7;44(5):606–15. doi: 10.1007/s00134-018-5148-2 (PMC6006241; doi:10.1007/s00134-018-5148-2)

## Supplemental Figures

**Supplementary Fig 1** Severity of illness using the ICNARC physiology score and (acute) 7-day mortality


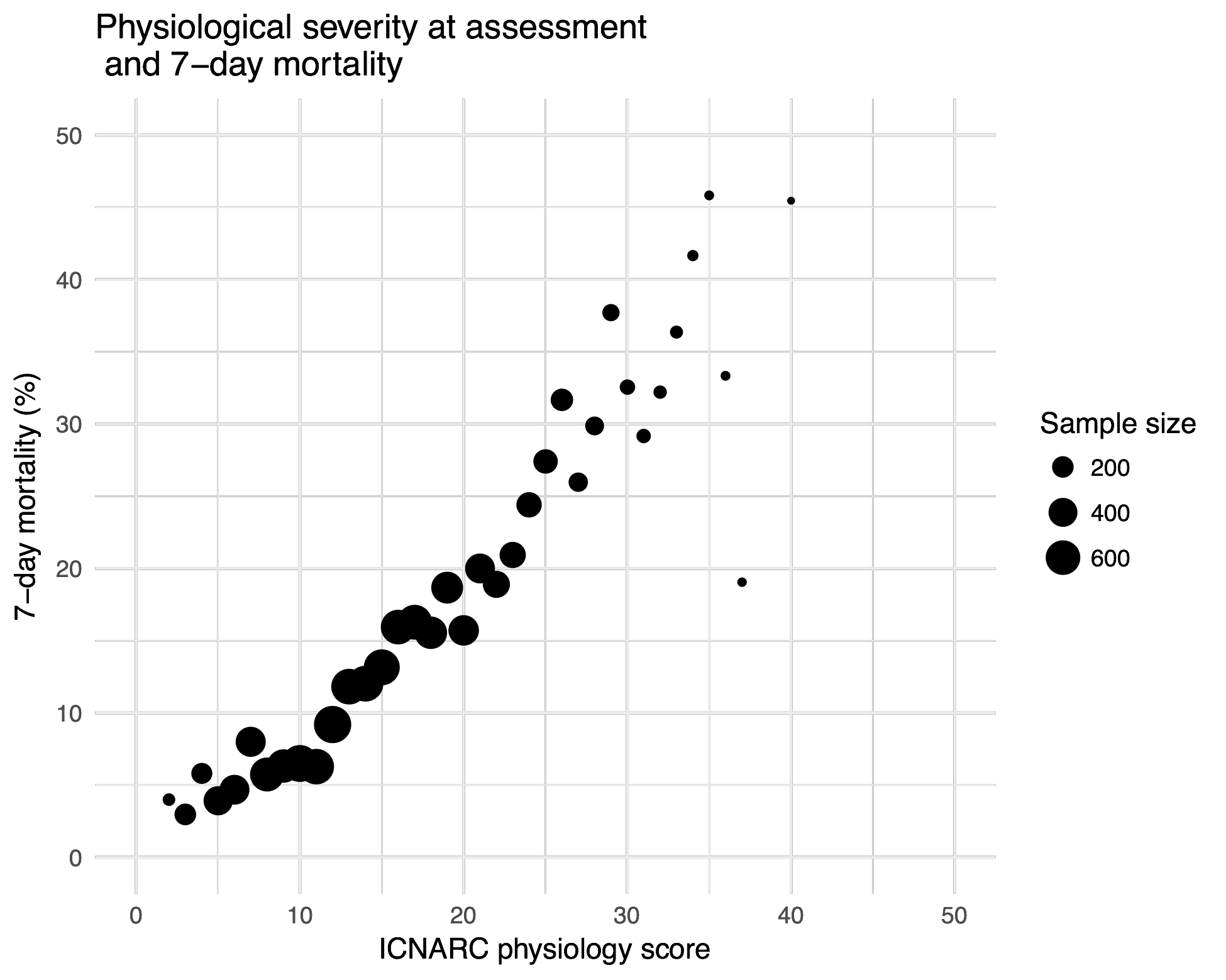


**Supplementary Fig 2** Time to admission to critical care: stratified by the critical care strain at the ward bedside assessment
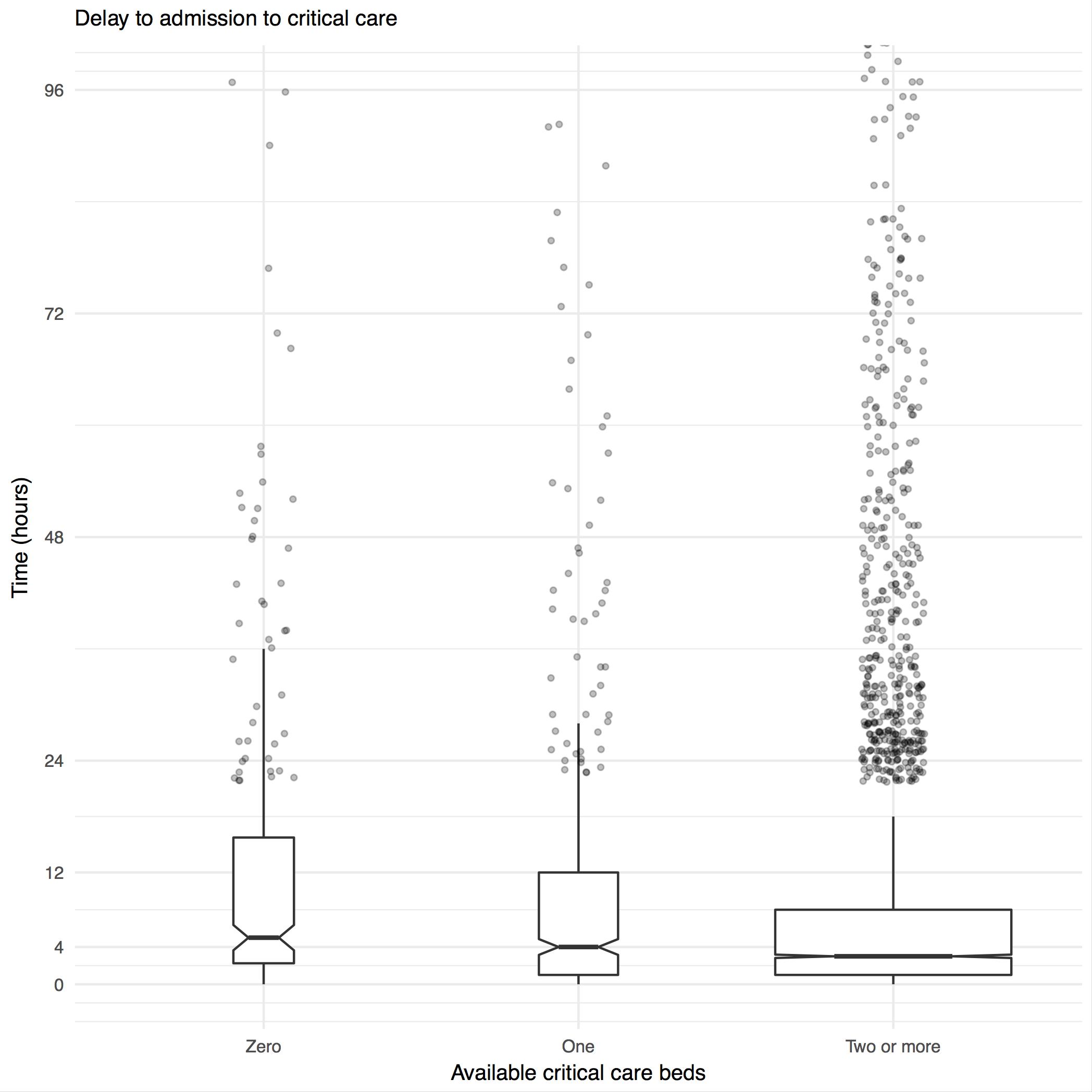


**Supplementary Fig 3** Patient disposition over time following bedside assessment for the subgroup recommended for critical care at bedside assessment: Proportion of patients currently in critical care, or dead without admission to critical care, and overall deaths by critical care unit strain at the time of the bedside assessment

**Supplementary Fig 4a and 4b** Analyses repeated using different coverage thresholds for considering data capture complete during the study. Data linkage rates between the (SPOT)light data and the ICNARC CMP data were used throughout the study to monitor quality. Where eligible admissions to a critical care unit were reported to the ICNARC CMP but not found in the (SPOT)light reports, then the concern was that ward referrals to ICU were not being captured. Hospitals were required to meet a minimum standard of 80% capture during the first three months, and, even after this period, those months where the data quality fell below this standard were also excluded. The unadjusted, risk adjusted, and instrumental variable analyses were therefore repeated using the additional data submitted that was 70-80% complete to - the ‘all’ data set. This included an additional 32 study months and 1013 patients. Similarly, the analysis was repeated amongst those hospitals meeting a higher 90% threshold to - the ‘best’ data set (43 hospitals, 210 study months, and 7076 patients). The mean monthly data linkage proportions in the ‘all’, ‘study’ and ‘best’ data sets were 92.9%, 94.6%, and 99·8%. Supplementary Figures 4a (all patients) and Figure 4b (subgroup recommended for critical care on assessment).


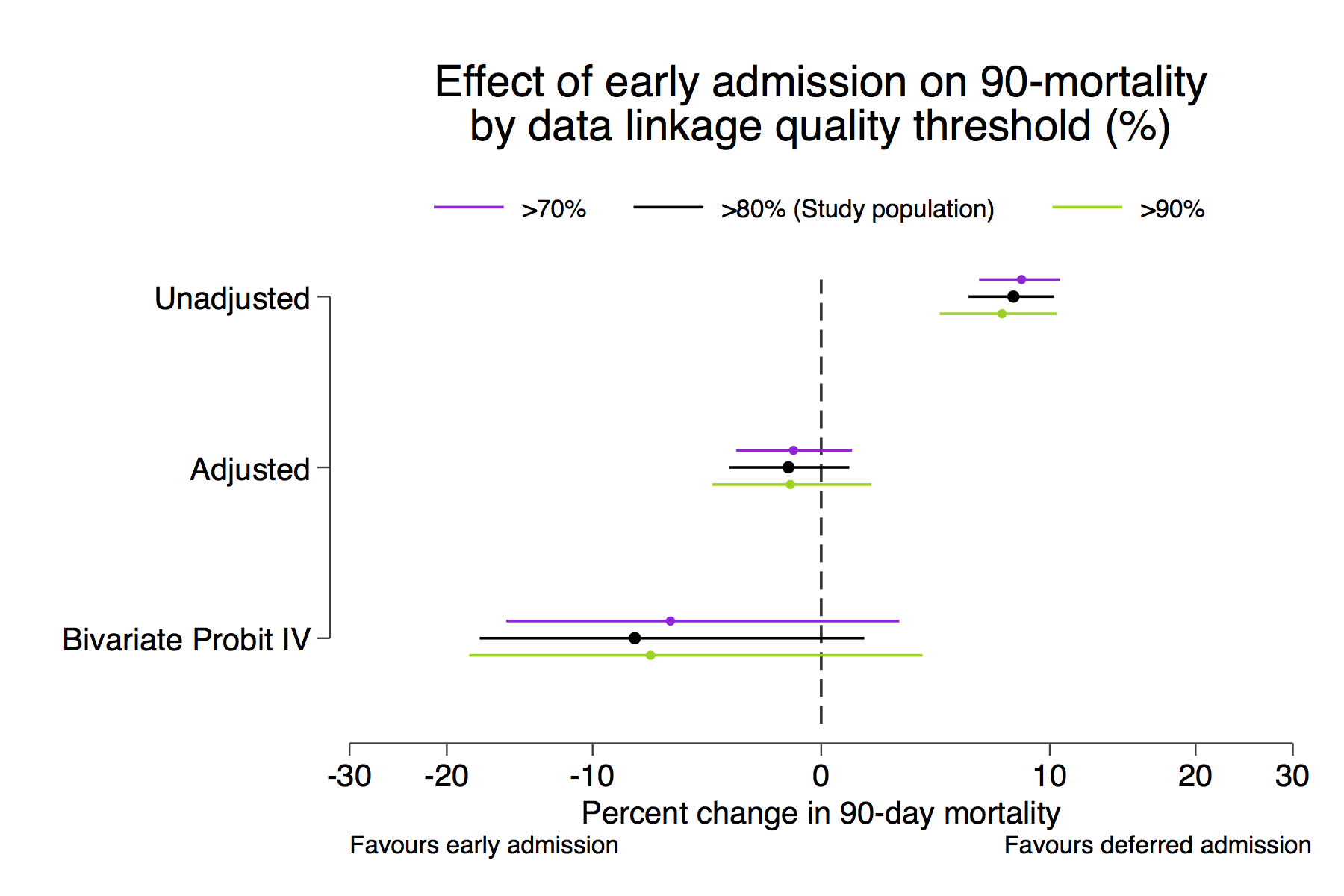


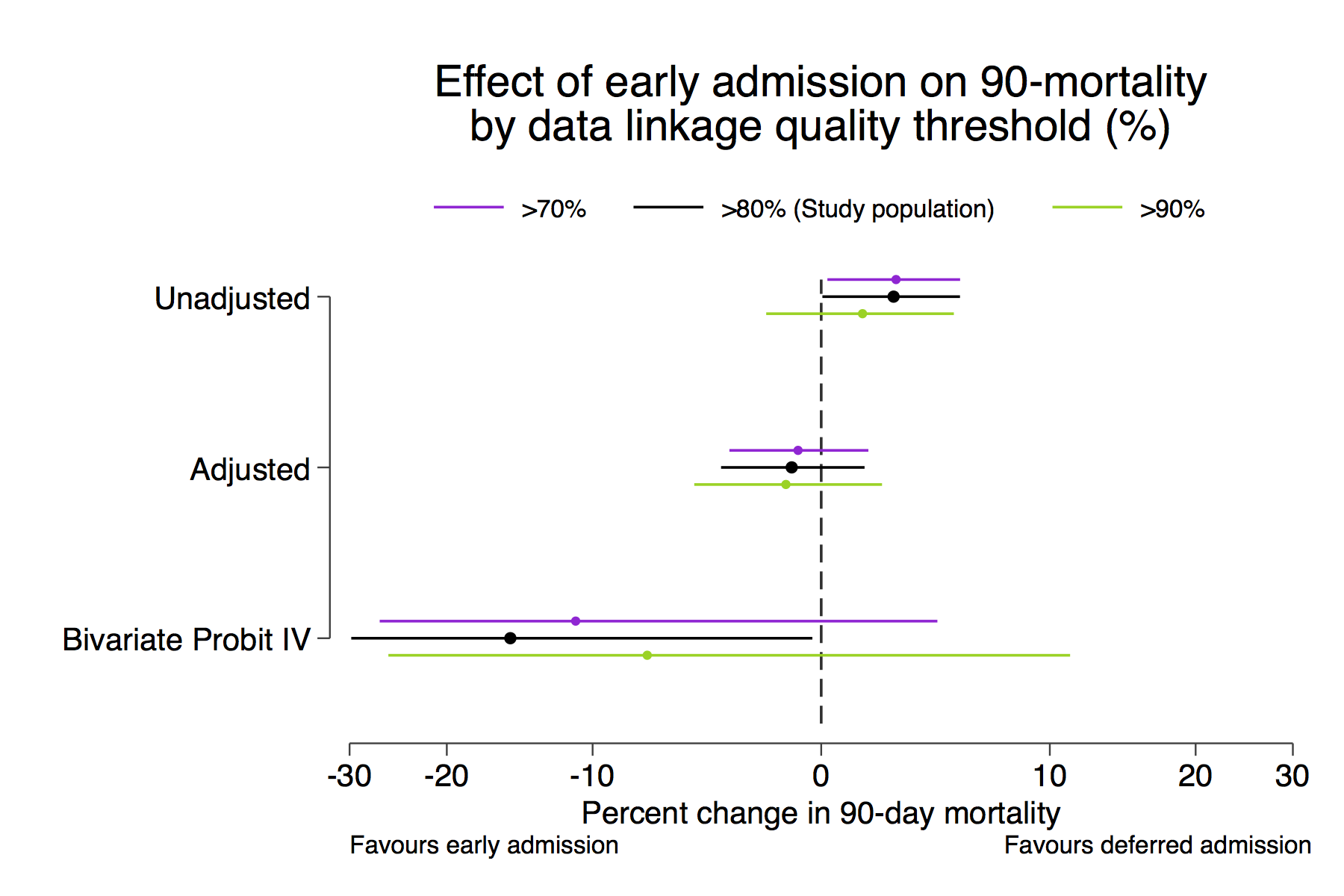


**Supplementary Fig 5** Baseline prognostic variables by critical care bed availability


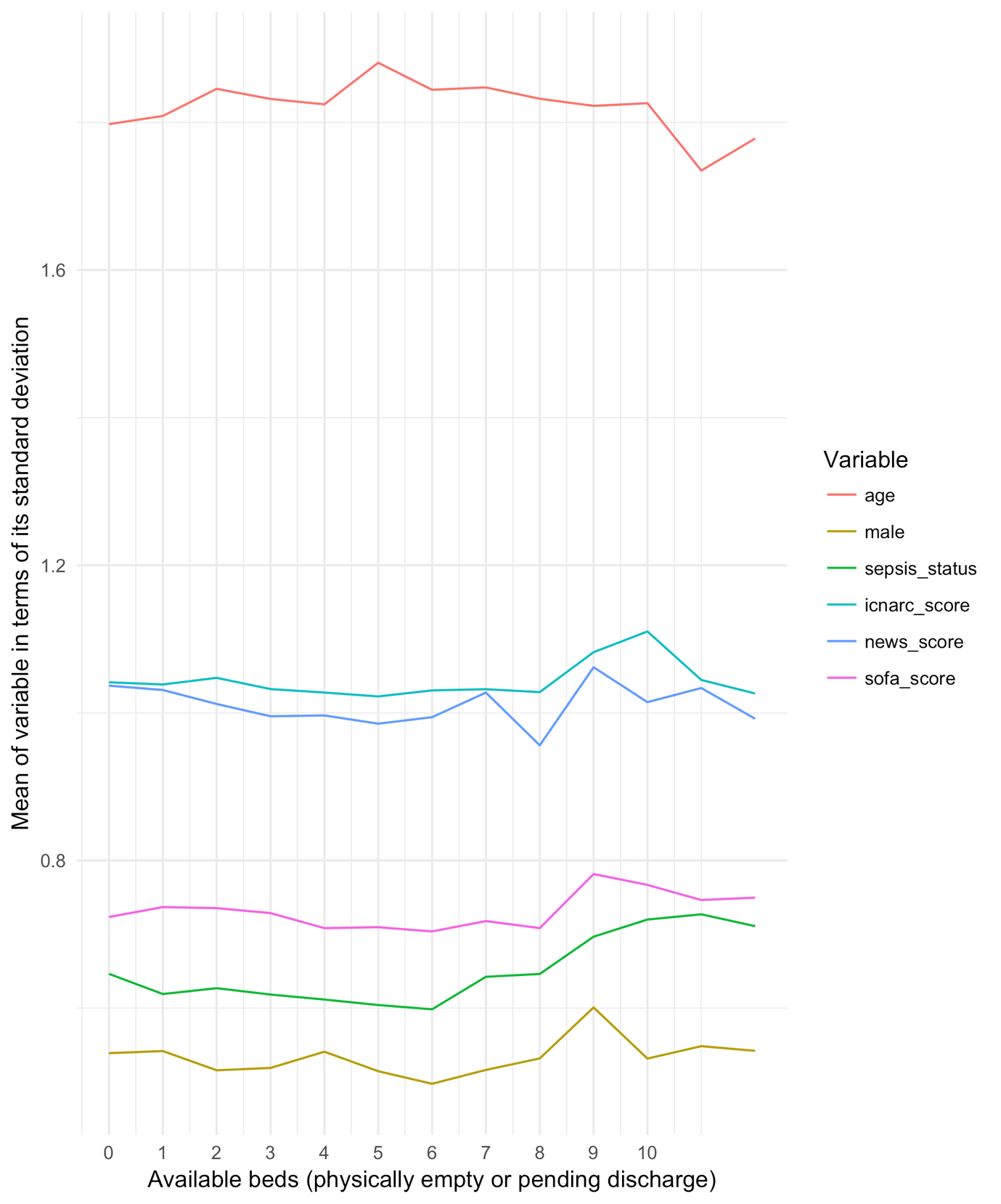

Supplement: Supplementary file 2 — Supplementary material 2 (DOCX 7702 kb) [file 134_2018_5148_MOESM2_ESM.docx]
