# Supplementary material for: Correction to: Impact on mortality of prompt admission to critical care for deteriorating ward patients: an instrumental variable analysis using critical care bed strain
Source: Intensive Care Med. 2018 Jun 8;44(5):701. doi: 10.1007/s00134-018-5254-1 (PMC6006216; doi:10.1007/s00134-018-5254-1)
Supplement: Supplementary file 1 — Supplementary material 1 (DOCX 28 kb) [file 134_2018_5254_MOESM1_ESM.docx]

# Supplementary materials - Acknowledgements

We wish to thank all the staff from all the sites and individuals that participated in the study.

## Participating sites

Airedale General Hospital; Arrowe Park Hospital; Barnsley Hospital; Basildon Hospital; Birmingham Heartlands Hospital; Blackpool Victoria Hospital; Bradford Royal Infirmary; Broomfield Hospital; Calderdale Royal Hospital; Castle Hill Hospital; Colchester General Hospital; Countess of Chester Hospital; Craigavon Hospital; Derriford Hospital; Dewsbury and District Hospital; Diana, Princess of Wales Hospital; Dorset County Hospital; Freeman Hospital; Furness General Hospital; Glan Clwyd District General Hospital; Harrogate District Hospital; Homerton Hospital; Huddersfield Royal Infirmary; Hull Royal Infirmary; Kent and Canterbury Hospital; Kettering General Hospital; King’s Mill Hospital; Lincoln County Hospital; Lister Hospital; Luton and Dunstable Hospital; Macclesfield District General Hospital; Maidstone Hospital; Medway Maritime Hospital; Musgrove Park Hospital; New Cross Hospital; Norfolk and Norwich University Hospital; North Middlesex University Hospital; Northampton General Hospital; Nottingham City Hospital; Pilgrim Hospital; Pinderfields Hospital; Poole Hospital; Queen Elizabeth Hospital Gateshead; Queen Elizabeth II Hospital; Queen Elizabeth, The Queen Mother Hospital; Queen’s Hospital, Romford; Queen’s Medical Centre; Rotherham General Hospital; Royal Berkshire Hospital; Royal Bolton Hospital; Royal Free Hospital; Royal Glamorgan General Hospital; Royal Hampshire County Hospital; Royal Preston Hospital; Royal Surrey County Hospital; Royal Victoria Hospital; Royal Victoria Infirmary; Scarborough Hospital; South Tyneside District Hospital; Southampton General Hospital; Southend University Hospital; Southport and Formby District General Hospital; St George’s Hospital; St James’s University Hospital; St Mary’s Hospital London; Stafford Hospital; Sunderland Royal Hospital; Tameside General Hospital; The Christie Hospital; The Ipswich Hospital; The James Cook University Hospital; The James Paget Hospital; The Princess Alexandra Hospital; The Queen Elizabeth Hospital, King’s Lynn; The Royal Blackburn Hospital; The Royal Liverpool University Hospital; The Royal London Hospital; Torbay Hospital; Tunbridge Wells Hospital at Pembury; Ulster Hospital; University College Hospital; University Hospital of North Staffordshire; Warrington Hospital; Watford General Hospital; West Cumberland Hospital; West Suffolk Hospital; Weston General Hospital; Wexham Park Hospital; Whipps Cross University Hospital; Whiston Hospital; Whittington Hospital; William Harvey Hospital; Worcestershire Royal Hospital; Wrexham Maelor Hospital; Wycombe Hospital; Wythenshawe Hospital; Yeovil District Hospital

## Research staff at participating sites

## Principal Investigators

## Dr David Higgins; Debbie Higgs; Dr Mark Holliday; Dr Richard Innes; Dr Ken Inweregbu; Dr V Jaidev; Dr Rajesh Jain; Dr Christina Jones; Dr Frieda Keane; Dr Anton Krige; Dr Ravindra Kumar; Dr Martin Kuper; Dr Shondipon Laha; Eleanor Lea; Dr Vanessa Linnett: Dr Jerome McCann; Professor Danny McCauley; Dr Michael Mercer; Dr Parvez Moondi; Dr Jeronimo Moreno Cuesta; Ruth Mullett; Nicky Newton; Dr Melanie Osborne; Dr Valerie Page; Kimberley Pallister; Dr James Pettit; Dr Barbara Phillips; Michele Platt; Catherine Plowright; Dr Richard Pugh; Sarah Quinton; Dr Ian Rechner; Dr Henrik Reschreiter; Jenny Ricketts; Dr Alistair Roy; Mrs Louise Ruigrok; Rajamani Sethuraman; Deborah Shaw; Professor Mervyn Singer; Dr Santokh Singh; Dr Jagtar Singh Pooni; Dr Chris Subbe; Dr Tamas Szakmany; Andrew Tillyard; Dr John Trinder; Dr Ioannis Tsagurnis; Michael Vangikar; Dr Kumaresh Venkatesan; Dr Grant Watling; Dr Philip Watt; Dr Rae Webster

## Research staff

## Myrtle Adoro; Dawn Adsetts; Mary Ann Ahaneku; Rebecca Ahern; Kirsty Ainsworth; Mike Albert; David Aldulaimi; Josephine Allen; Karine Anderson; Alison Anstead; Karen Apps; Diane Arnold; Liz Arthan; Jennifer Assimakopoulos; Hannah Bagshaw; Derek Bainbridge; Melissa Balcorta; Jacqueline Baldwin; Lee Barnes; Helena Barnes; Chris Barton; Dawn Bayford; Deborah Bayoan; Zoe Beardow; Heather Bebbington; Mary Beech; Dr Martin Beed; Bernardette Beirne; Stephanie Bell; Michelle Bellamy; Geeta Bhaggan; Michele Bianchi; Isobel Bird; Christine Blacklock; Dr Mark Blunt; Clare Bolger; Dr Stephen Bonner; Claire Bonner; Ben Booth; Ian Bottomley; Ellen Bowley; Peggy Bradley; Sian Bradley; Aiden Brennan; Gillian Bridges; Yvette Brigdale; Spike Briggs; Maria Britton; James Bromilow; Lauren Brotherston; Christina Browett; Leanne Brown; Anne Budge; John Burke; Chris Burnett; Mary Burt; Michelle Butt; Ann Cadd; Donna Calvert; Lily Capili; Michelle Carey; Mark Carpenter; Christine Carroll; David Cartlidge; Tina Cassidy; Tracy Cathcart; Louise Cawthorn; Shibu Chacko; Wendy Chamberlain; David Chapman; Julia Charsley; Andrew Chatwin; Debra Cheetham; Shanti Chelliah; Harnita Chohan; Rachel Clarkson; Carly Claxton; Dr Ian Clement; Louise Clements; Julie Cocker; Rory Collier; Rosie Corbett; Richard Corderoy; Dr John Criswell; Tina Crossey; Wendy Crow; Lesley Cruse; Daniel Cummings; Dr Jason Cupitt; Lynn Cushing; Sandra D'Arcy; Julie Darroch; Deborah Dawson; Rachael Day; Nilofer Dayal; Kim de Courcy-Golder; Gabrielle De Selincourt; Dr Simon Deacock; Caroline Dear; Gonzalo Delacerda; Grace Delaney-Segar; Pauline Denness;

## Margaret Mary Devaney; Emma Devlin; Sandeep Dhir; Christine Dickson; Angela Ditchfield; Mabandla Dlamini; Nicola Doherty; Siobhan Doolan; Sarah Dow; Allyn Dow; Susan Dowling; Elaine Duncan; Ruth Dunne; Simon Dyer; Margaret Eaton; Becky Edwardes; Mary Edwards; Ed Ekanem; Sue Elderton; Lee Ellis; John Elmore; Mark Emmott; Rachel English; Gillian Errington; Nigel Evans; Andrew Evans; Chloe Evans; Alan Fairclough; Frances Farnworth; Maria Faulkner; James Featherstone; Aoife Fitzgerald; Dawn Marie Forrester; Laura Foster; Karen Francis; Jayne Fraser; Sally Fray; Emma Fryatt; Jitendra Garg; Georgia Gately; Dr Emma Gent; Karin Gerber; Tanya Gill; Neil Glendinning; Georgina Glister; Josie Goodsell; Dr Shameer Gopal; Dr Andy Gosling; Mark Gotecha; Denise Graham; Ken Grainger; Lynn Gregg; Emma Gregson; Michael Gribbon; Amanda Griffiths; Patricia Guilfoyle; Janette Guymer; Samantha Hagan; Prema Hall; Angela Hall; Andrew Hall; Diana Hardy; Donna Harrison-Briggs; Jillian Hartin; Janice Hartley; Lesley Hawkins; Rhona Hayden; Karen Hayden; Elizabeth Haynes; Dr Marie Healy; Anne-Marie Heath; Sheila Hennessy; Bernardo Hernandez; Jackie Hewlett; Charley Higham; Karen Hill; Ashley Hilliard; Paul Hodge; Karen Hogben; Dr Douglas Holden; Lai Holden; Dr Rachael Homer; Marion Hood; Elspeth Horner; Stephanie Houlker; Vladimir Hudak; Thomas Hughes; Keith Hugill; Sally Humphreys; Julian Hunt; Elaine Hunter; Alan Hurding; Will Huxham; Tahir Idrees; Ursula Igbokwe; Clare Jackson; Lee-Ann Jacob; Reni Jacob; Nicola Jacques; Alicia Jakeman; Nesta James; Marta Januszewska; Fiona Jefferies; Marghanita Jenkins; Beverley Jobson; Martin Johnson; Susan Johnson; Rachel Jolly; Ian Jones, Ronald Jones; Carys Jones; Craig Jones; Anne Joy; Dr Agilan Kaliappan; Victoria Katsande; Sarah Keeling; Andrea Keenan; Carys Keenleside; Karen Kelly; Susan Kelly; Nicci Kelsall; Jillian Kelsall; Sarah Kempster; Maureen Kermack; Dawn Killeen; Sheila King; Karen Kinnear; Katherine Kite; Andy Kong; Yasmine Koodun; Grace Lacaden; Sarah Lafbery; Stephen Laver; Leanne Lawson; Dr Robert Lewis; Dr Jeffrey Little; Angela Locke; Christopher Loew; Sarah Long; Joanne Lowther; Matthew Ludford; Nuru Lushpay; Michael MacAndrew; Elizabeth Magee; Karen Magill; Samuel Magombe; Sean Mahoney; Janine Mair; Dr Paul Mallett; Nick;y Marks; Gail Marshall; Debbie Marshall; Dr Terry Martin; Claire Martin; Jane Martin; Heather Mason; Roslyn Mattukoyya; Chris May; Shaun McAuliffe; Amanda Jane McCairn; Debbie McCall; Joanna McCormick; Ann McGinley; James McGowan; Karen McGuire; Diana McHale; Anthony McKay; Dr Raymond McKee; Helen McMillan; Robert McMonagle; Denise Melling; Johannes Mellinghoff; Pauline Mercer; Colin Merrill; Nicola Metcalfe; Diane Miller; Wendy Miller; Nick Millett; Christine Mitchell-Inwang; Kate Molloy; Sarah Moreton; Kathryn Morgan; Monica Morosan; Anna Morris; Chris Morrissey; Lucy Mummery; Pamela Munro; Dr Y Myint; Sue Naidoo; Dr Abdul Nazar; Tim Needham; Karen Neville; Alyson Newlove; Barry Newman; Khanyi Nhamburo; Lillian Norris; Dr Andrew Norton; Pippa Norton; Simon Nourse; Ingrid O'Neil; Andy Padkin; Sheik Pahary; Natascia Parry; Deborah Paton; Heather Payne; Lucy Pearman; Dr Rupert Pearse; Maggie Peat; Emma Pelech; Professor Gavin Perkins; Angela Peskett; Hannah Phillips; Julie Phillips; Yvonne Picking; Nuno Pinto; Jessica Piper; Sarah Pitt; Vivienne Pixton; Julie Platten; Jennifer Plume; Una Poultney; Erin Povey; Valerie Powell; Dr Ken Power; Jackie Power; Sioned Price; Elizabeth Prince; Tina Pritchard; Andrew Quinn; Tracy Quintrell; Dr James Raitt; Parizade Raymode; Dr David Redman; Jevangie Regalado; Katie Reid; Karen Rhodes; Dr Lesley Rice; Pippa Richards; Jenny Ritzema; Jason Roberts; Helen Robertson; Dr Nicole Robin; Gill Robinson; Hazel Robinson; Sarah Robinson; Kath Robinson; Victoria Robson; Tracey Robson; Natalie Rogers; Andrew Rooks; Melissa Rosbergen; Julie Rugg; Gill Runkee; Mandy Russell; Wendy Sandajan; Glenn Saunders; Jane Saunders; Claire Scott; Deepti Sebastian; Bibi Ashmin Sham; Victoria Sharman; Julie Sharpley; Simon Shaw; Jane Shewan; Kim Siggers; Linda Sime; Anthony Simpson; Mohan Sivarajaratnam; Ben Skinner; Kerry Slaney; Lynne Slater; Tracy Slater-Hawksworth; Christopher Smalley; Neil Smith; Sue Smolen; Ali Spencer; Hazel Spencer; Dr Tom Stambach; Caitriona Stapleton; Linda Stephenson; Lorraine Stephenson; Ruth Stewart; Gustav Strandvik; Karen Swan; Mandy Swanepoel; Carin Swanevelder; Serena Swanston; Kate Tantam; Helen Taylor; Fiona Teeling; Dr Richard Thomas; Andy Thorn; Janet Thornberry; Clare Thorogood; Orla Thunder; Andrew Timmins; Lucy Tindall; Gillian Tomlinson; Joanne Topliffe; Christina Towers; Dawn Trodd; Andrea Turner; Genny Turner; Freya Velzeboer; Christine Vickery; Samuel Waddy; Marianne Wain; Dr Richard Walker; Roy Walker; Rachel Walker; Anna Walker; Jo Walker; Adam Walker; Angela Walsh; Helena Walsh; Dr Geoff Watson; Alison Welburn; John Welch; Professor Ingeborg Welters; Ross Whetton; Michael White; Griania White; Nicola Whittaker; Andrea Whitton; Desmond Wilkinson; Anne Williams; Steven Williams; Sheila Williams; Nicky Williams; Robert Williams; Sarah Williams; Karen Williams; Barbara Williams-Yesson; Caroline Wilson; Julie Wilson; Dr Steve Wimbush; Sarah Wimpenny; Kate Wong; Rachael Worton; Caroline Wrey Brown; Dr Stephen Wright; Abigayle Wright; Professor Ian Young; XiaoBei Zhao; Michelle Zhao

## Study Steering Group

Independent members:

Dr Kath Daly; Professor Nick Mays; Dr Ann McDonnell; Professor Gary Smith; Dr Chris Subbe; John Welch

Non-independent members:

Professor David Harrison; Dr Steve Harris; Professor Kathy Rowan; Dr Colin Sanderson; Professor Mervyn Singer
